# Resonance‐Enhanced Multiphoton Ionization Spectroscopy of Monocyclic and Polycyclic Aromatic Hydrocarbons in the Gas Phase

**DOI:** 10.1002/rcm.10096

**Published:** 2025-06-21

**Authors:** Carolin Schwarz, Fabian Etscheidt, Christian Gehm, Johannes Passig, Sven Ehlert, Thorsten Streibel, Ralf Zimmermann

**Affiliations:** ^1^ Joint Mass Spectrometry Centre/Chair of Analytical Chemistry University of Rostock Rostock Germany; ^2^ Leibniz Institute for Baltic Sea Research Warnemünde Rostock Germany; ^3^ Photonion GmbH Schwerin Germany; ^4^ Department Life, Light & Matter (LLM) University of Rostock Rostock Germany; ^5^ Joint Mass Spectrometry Centre, Cooperation Group “Comprehensive Molecular Analytics” (CMA) Helmholtz Zentrum München Neuherberg Germany

**Keywords:** (polycyclic) aromatic hydrocarbons ((P)AH), optical parametric oscillator (OPO), resonance‐enhanced multiphoton ionization (REMPI), spectroscopy, time‐of‐flight mass spectrometry (TOFMS)

## Abstract

**Rationale:**

Aromatic hydrocarbons (AHs) and polycyclic aromatic hydrocarbons (PAHs) pose significant risks to human health and the environment due to their toxic and carcinogenic properties. These depend strongly on molecular structure, with even isomers exhibiting different characteristics. Consequently, when conducting a risk assessment of a sample, a rapid and reliable detection technique capable of differentiating between isomers is crucial.

**Methods:**

Time‐of‐flight mass spectrometry (TOFMS) combined with (1 + 1) resonance‐enhanced multiphoton ionization ((1 + 1)‐REMPI) has proven to be a promising approach due to its wavelength selectivity for different structures. An optical parametric oscillator generated UV radiation from 213 to 300 nm from the third harmonic (355 nm) of a Nd:YAG laser beam. A thermogravimetric system was applied to transfer the substances into the gas phase.

**Results:**

We performed REMPI spectroscopy of 48 monocyclic and polycyclic aromatic hydrocarbons, including compounds with various substituents (alkyl groups, ‐OCH_3_, ‐SH, ‐OH, ‐Cl) and heteroatoms (N, O, S). The observed spectral shifts correlate with ring number as well as the type, number, and position of substituents and heteroatoms. While these shifts are comparable to trends observed in absorption spectra, variations in intensity arise due to differences in excited‐state lifetimes and the cross sections of both absorption steps. It was further demonstrated that the selected wavelength range, extending to a lower limit of 213 nm, is especially beneficial for the naphthalenes. The relative photoionization cross sections of the investigated compounds have been calculated, showing that the aforementioned structural dependencies also influence the ionization efficiency.

**Conclusions:**

In common applications, these results may be used to determine a suitable laser wavelength for the substances of interest in order to achieve a higher level of sensitivity. For tunable laser applications, they serve as a reference for distinguishing and quantifying isomers in complex mixtures based on spectral shifts.

## 1 Introduction

Aromatic hydrocarbons (AHs) and polycyclic aromatic hydrocarbons (PAHs) are emitted by incomplete combustion processes and pose a threat to humans and the environment due to their toxic and carcinogenic properties [1, 2]. The degree of toxicity is dependent upon the chemical structure of the molecule, and even isomers may exhibit different characteristics [3]. Consequently, for an accurate toxicity assessment of environmental samples, the precise identification of AHs and PAHs, as well as the differentiation of isomers, is essential.

For this purpose, the combination of resonance‐enhanced multiphoton ionization (REMPI) with a time‐of‐flight mass spectrometer (TOFMS) has proven to be an effective approach, offering high sensitivity and selectivity. Due to the soft character of REMPI in comparison to other ionization techniques, such as electron impact (EI), the fragmentation of molecules can be mostly excluded [4]. Moreover, its capability for real‐time measurements makes REMPI‐TOFMS particularly well suited for the online analysis of complex samples [5]. The most straightforward approach to perform REMPI involves the use of two photons of identical wavelength, a process referred to as (1 + 1)‐REMPI. It is limited by the combined energy of the two photons, which needs to be higher than the ionization energy. In this process, the first photon is absorbed by the molecule, resulting in the transition of an electron to an excited state. During the lifetime of the excited state, a second photon must be absorbed to induce ionization.

As a result, REMPI is a wavelength‐dependent process, as the energy of the excited states depends on the chemical structure of the compounds. Therefore, the knowledge of the specific REMPI pattern of each substance could enable the identification of these substances and the differentiation between isomers. Consequently, the combination of a tunable UV laser system with TOFMS might offer two distinctive advantages: the potential identification of substances based on their ionization pattern and an enhanced sensitivity by adjusting the laser wavelength to the substance of interest.

REMPI spectra generally resemble absorption spectra, thus analogous trends related to ring number, annellation, and the type and position of substituents are anticipated. However, while absorption spectra provide a general indication of excitation wavelengths, they do not necessarily predict optimal REMPI conditions. REMPI spectra are significantly influenced by factors such as lifetimes of excited states and ionization cross‐sections, which cannot be directly inferred from absorption data alone [6, 7]. Therefore, direct REMPI measurements are crucial for selecting optimal ionization wavelengths and improving analytical performance.

Studies by Haefliger et al. [6] and Carpentier et al. [8] have focused primarily on the REMPI spectra of parent AHs and PAHs, presenting only a few spectra of their derivatives in complex samples. However, the derivatives are often considered to be more toxic and therefore highly relevant for assessing health risks associated with environmental pollutants, such as engine exhaust and cigarette smoke [9, 10]. For this reason, they are the main subject of this work. To systematically investigate the impact of structural modifications on REMPI spectra, we performed REMPI spectroscopy on 48 AHs and PAHs within a wavelength range of 213 to 300 nm. In general, REMPI intensities are higher for bands at shorter wavelengths [7], highlighting the importance of an extended wavelength range, particularly for substances with absorption bands in this region. The measurements were conducted on pure substances, which were volatilized using a thermogravimetric system. In order to quantify the ionization process, we determined relative photoionization cross sections (relPICS) at 213, 248, and 266 nm and the peak maximum wavelength (λ_max_) for each substance, using toluene as a reference.

## 2 Experimental Section

### 2.1 Sample Introduction

In our study, we investigated the REMPI spectra of 48 substances, including monocyclic and polycyclic aromatic hydrocarbons with different substituents and several heterocyclic compounds. Each substance was evaporated individually in the thermogravimetric system (TG; STA7200 RV from Hitachi High‐Tech Corp., Japan). A constant gas stream was produced by heating to a specific temperature. The choice of temperature was a combination of generating a signal high enough to produce a sufficient signal and low enough not to saturate the TOFMS detector. By providing a nitrogen flow of 200 mL/min, the molecules were transported from the TG into a fused silica capillary (ID = 250 μm). The capillary was placed in a heated transfer line (265°C). The molecules were introduced into a TOFMS (RFT10, Stefan Kaesdorf GmbH, Germany). A detailed description of the setup of the mass spectrometer can be found in Mühlberger et al. [11].

### 2.2 Laser Setup

**FIGURE 1:**
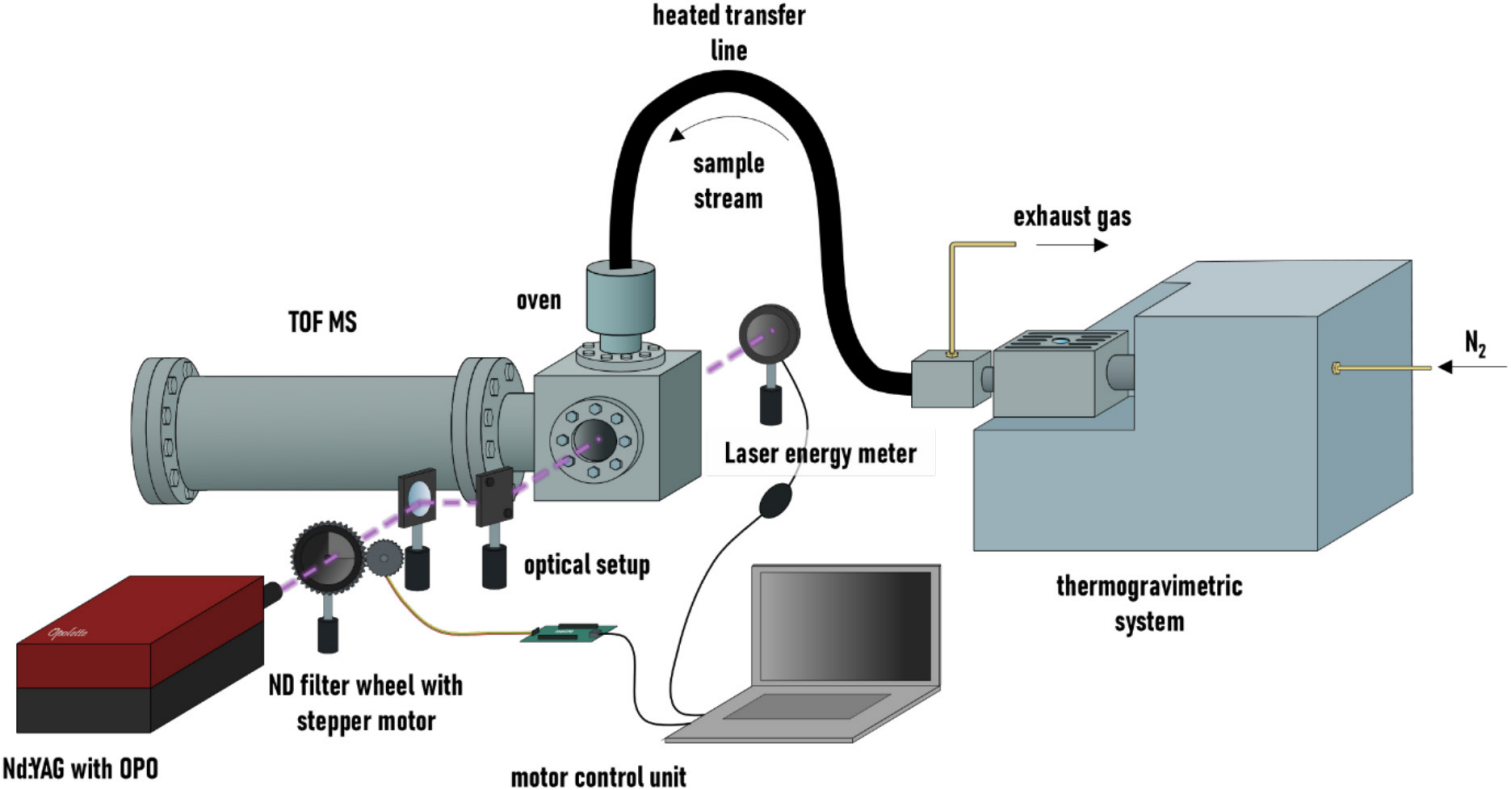
Schematic experimental setup with TOFMS, tunable UV laser (Nd:YAG with OPO), thermogravimetric system, and the laser beam path.

An optical parametric oscillator (OPO) pumped by a Nd:YAG laser (Opolette 355 LD w/UV from OPOTEK LLC, US) was used to generate the UV radiation for the ionization (see Figure 1). This tunable laser system is able to produce UV radiation in a wavelength range between 210 and 410 nm. For the targeted substances and the selected ionization method (1 + 1)‐REMPI, a wavelength range between 213 and 300 nm was considered sufficient. The produced wavelength has been monitored with an internal spectrometer and does not deviate more than 0.05 nm (linewidth: 4–7 cm^−1^, pulse width: 7 ns) from the desired wavelength. While scanning through the wavelength range, an internal beam path change in the Opolette system at 255 nm required a subtle realignment of the beam towards the TOFMS. The beam diameter when entering the TOFMS was 4 mm.

To compensate for wavelength‐dependent changes in the pulse energy, the energy had to be adjusted to achieve the same fluence at each wavelength. An energy sensor (PE50‐C, Ophir Optronics Solutions Inc., Israel) was placed behind the ion source, and a circular, continuously variable reflective neutral density filter (NDC‐50C‐2M from Thorlabs Inc., USA) was placed in front of the ion source. By using a custom LabVIEW PID controller script with a stepper motor controlled by an Arduino Mega microcontroller board (Arduino S.r.l., Italy) connected to the filter wheel, we were able to automatically adjust the filter wheel and control the energy, thus keeping the energy constant over the various wavelengths. A more detailed description of the energy control system can be found in [12]. After passing the filter, the laser beam was directed into the ion source via two mirrors (UV Enhanced Aluminum, wavelength range 200–700 nm, Newport Corp., USA). On entering and exiting the ion source, it passed two UV fused silica windows (entrance: thickness 10 mm, diameter 50 mm; exit: thickness 3 mm, diameter 25 mm, UQG Optics, UK). REMPI spectra were obtained by scanning through the above wavelength range with a step size of 0.5 nm. The energy was thereby regulated to 50 μJ. Each wavelength was measured with 200 laser shots, and each scan was performed three times.

### 2.3 Data Handling

Due to the fact that the PID controller needed some time to adjust to the correct position for the desired energy, the first 80 laser shots were excluded from the calculations. For normalization, we made sure that the dependence between TOFMS signal intensity and mass loss was linear. The data had subsequently been normalized over the concentration. Finally, the spectra were averaged across three measurements, and the resulting mean spectrum was used for the data presented in this paper.

## 3 Results and Discussion

### 3.1 Parent PAHs

In addition to the REMPI spectra provided by Haefliger et al. [6] and Carpentier et al. [8], absorption spectra were employed for the validation of the methodology. This allows for the identification of the corresponding absorption bands. However, it should be noted that the majority of absorption spectra documented in the literature have been obtained from substances in solution, which may result in solvent effects that could potentially shift the spectra by several nanometers in comparison to the data presented in this study [13].

**FIGURE 2:**
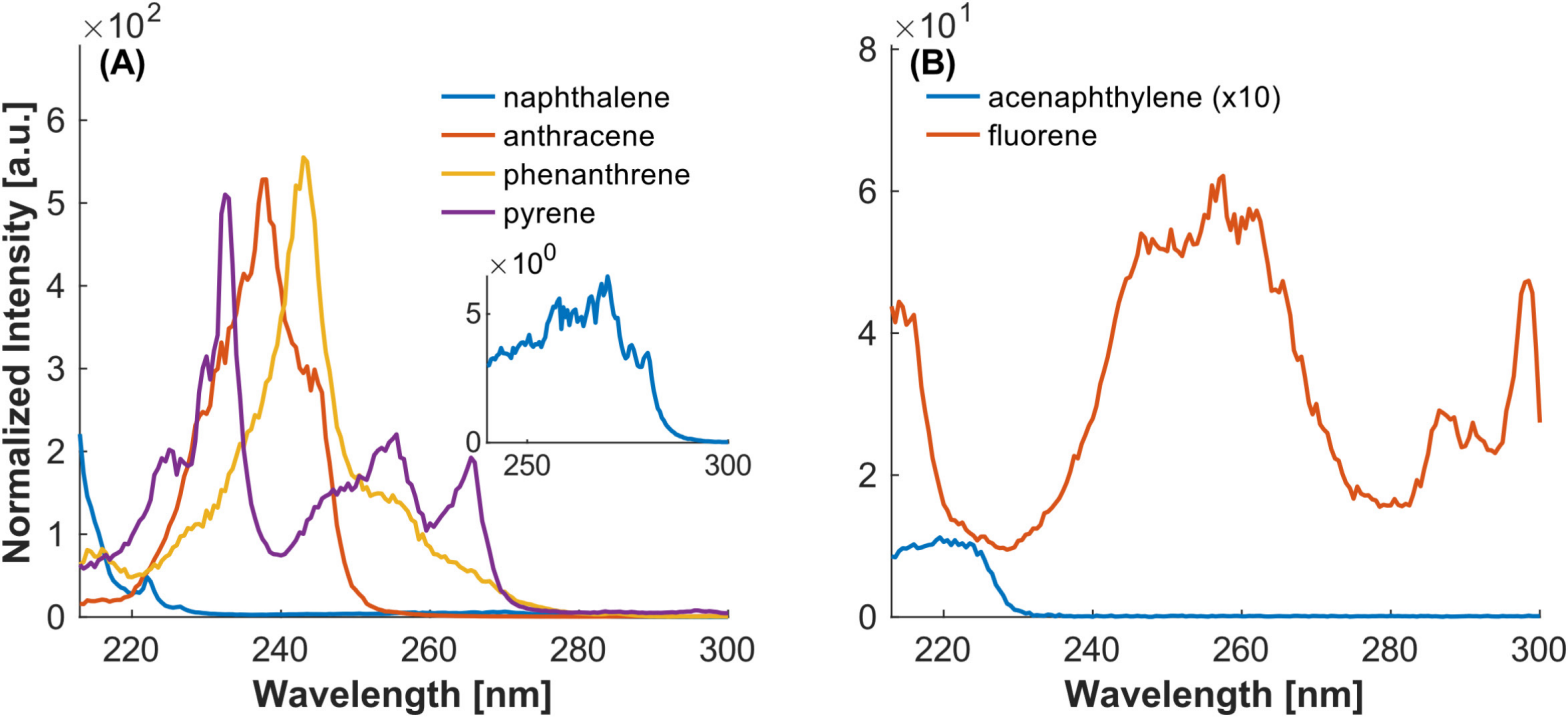
REMPI spectra of parent PAHs. (A) Only six‐membered ring containing PAHs. (B) Five‐membered ring containing PAHs. Intensities normalized to the maximum of toluene.

Figure 2A presents the spectra of naphthalene, anthracene, phenanthrene, and pyrene. As discussed by multiple authors [14, 15], an increase in ring number results in extended conjugation, which causes a bathochromic shift (shift towards longer wavelengths). While this shift is evident in all compounds with higher ring numbers compared to naphthalene, it is crucial to acknowledge the significant influence of ring annellation on the position of the band. When the annellation is not linear, as in phenanthrene or pyrene, this shift can be observed for the S_1_ states, but not for the higher excited states recorded in the REMPI spectra.

According to Salama et al. [16], the primary absorption band of naphthalene at wavelengths below 213 nm corresponds to the S_3_ electronic transition, whereas the maximum absorption of anthracene at 238 nm is attributed to its S_4_ transition [17]. In contrast, pyrene, which consists of four fused benzene rings, but in a nonlinear arrangement, exhibits its main absorption at 232.5 nm, assigned to the S_4_ state [18].

Phenanthrene, due to its angular annellation, exhibits increased stability relative to anthracene, a property commonly associated with a higher ionization energy and, consequently, a hypsochromic shift (shift towards shorter wavelengths). However, the REMPI spectra reveal a bathochromic shift of the dominant peak at 243 nm, ascribable to the S_5_ transition of phenanthrene [19]. This phenomenon can be attributed to a disparity in the distribution of bands associated with the angular arrangement.

The REMPI spectra of some five‐membered rings containing PAHs are displayed in Figure 2B. Previous investigations of absorption spectra have demonstrated that the presence of a five‐membered ring is accompanied by a bathochromic shift [15]. In accordance with this observation, acenaphthylene demonstrates a redshift in comparison to naphthalene, a finding that has previously been documented in the absorption spectra reported by Liu et al. [20]. At the same time, the signal intensity decreases. Other excited states, as observed in the absorption spectra, were not found in the REMPI spectrum.

The REMPI spectrum of fluorene exhibits four distinct bands, which are in agreement with the excited states reported by Bree et al. [21]. According to their analysis, the broad peak around 255 nm corresponds to the S_3_ transition, while the peak below 220 nm is attributed to the S_4_ state. In general, it can be stated that the spectra of the parent PAHs are consistent with data from the literature [6, 8], thus confirming their results.

### 3.2 Substituted AHs and PAHs

Adding substituents to the parent aromatic structure in general can cause two effects. By adding an auxochrome (electron donating group) to the chromophore, which causes a +I or +M effect, typically a bathochromic and hyperchromic shift (increase in intensity) is induced. Conversely, the addition of an antiauxochrome (electron‐withdrawing group) results in a hypsochromic and hypochromic (reduction in intensity) shift by causing a ‐I or ‐M effect [22, 23]. The following discussion will focus on the addition of several auxochromes (OH, OCH_3_, alkyl groups, SH, Cl).

#### 3.2.1 Alkylated AHs

**FIGURE 3:**
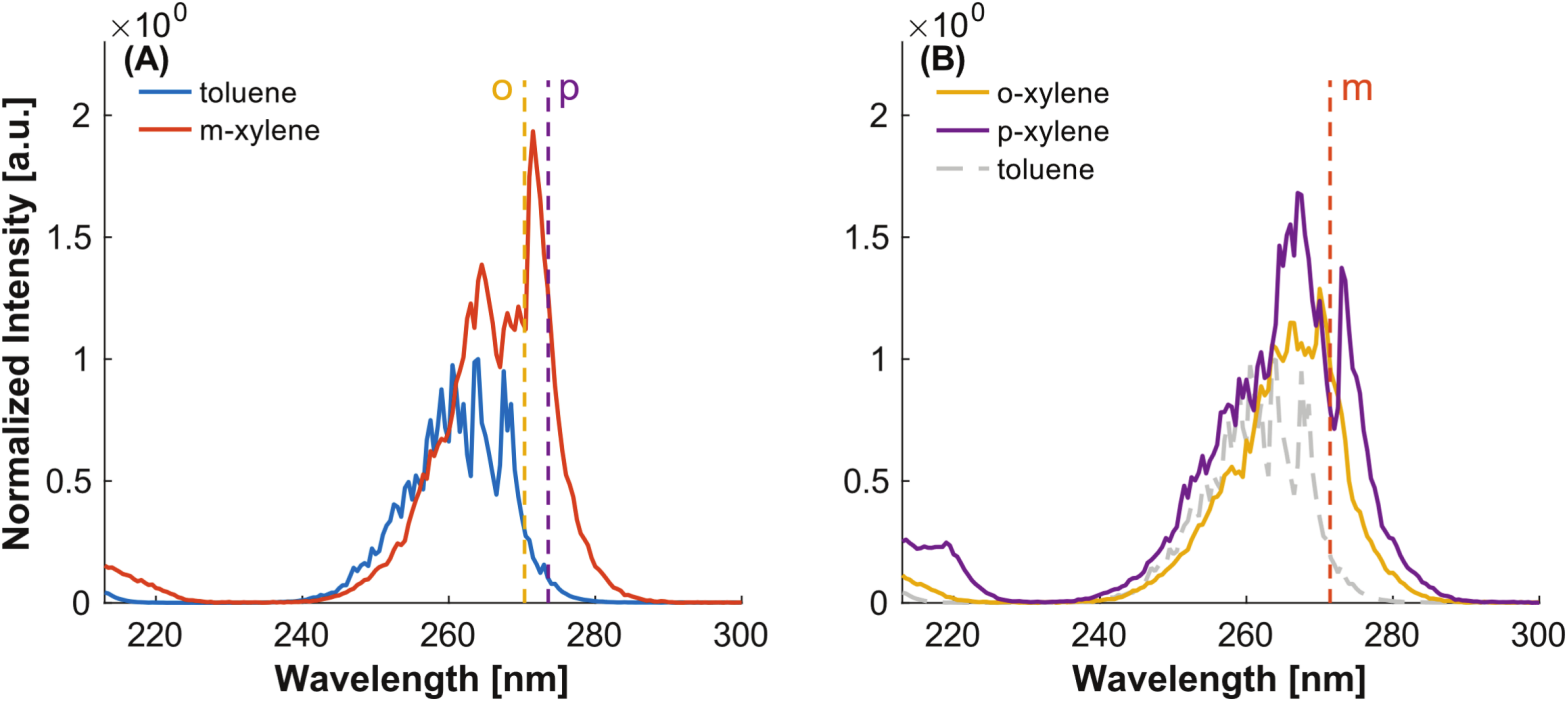
Investigation of the influence of number and position of alkyl groups. (A) Toluene, containing one methyl group, and meta‐xylene, containing two methyl groups, with shifts of the distinctive band at around 270 nm of ortho‐ and para‐xylene for better comparability. (B) Ortho‐ and para‐xylene with the shift of meta‐xylene for better comparability. Intensities normalized to the maximum of toluene.

The simplest representative of alkylated benzenes is toluene, containing one methyl group. According to Carpentier et al. [8] its spectrum is shifted by approximately 8 nm towards red compared to benzene. Its REMPI spectrum shows an accumulation of peaks between 250 and 270 nm (see Figure 3A), which can be assigned to the S_1_ state [24]. The recorded REMPI spectrum is also in very good agreement with data from the literature [8], validating the findings obtained from complex samples.

Adding more alkyl groups typically results in a bathochromic shift. This is due to the inductive effect of the alkyl groups, which increases the energy of the ground state, as shown in [25, 26]. Consequently, a redshift can be observed from toluene via xylene to trimethylbenzene (TMB).

For xylene, a bathochromic shift of o‐xylene < m‐xylene < p‐xylene was observed (Figure 3B). Thus, not only the number of alkyl groups but also their position influences the spectrum. The closer the methyl groups are to each other, the higher the steric hindrance between them. Steric hindrance reduces the electronic interaction between alkyl groups and the benzene ring, thereby causing a shift in the spectrum towards shorter wavelengths, typically accompanied by a hyperchromic shift [27].

**FIGURE 4:**
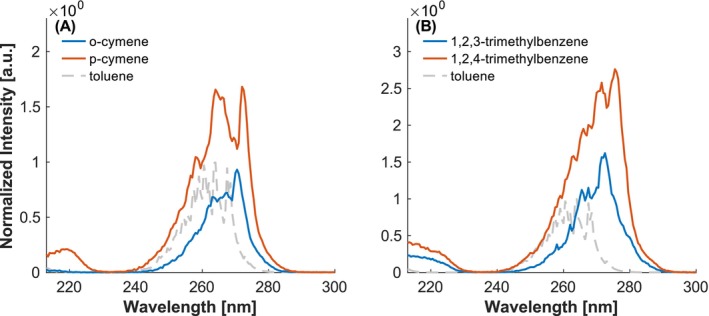
Investigation of the influence of number and position of alkyl groups. (A) Ortho‐ and para‐cymene, containing one methyl and one isopropyl group. (B) Two TMBs with methyl groups in different positions. Intensities normalized to the maximum of toluene.

The same effect is observed for both cymene and trimethylbenzene, where p‐cymene exhibits a greater redshift and higher intensity than o‐cymene, and 1,2,4‐TMB shows a more pronounced shift compared to 1,2,3‐TMB (Figure 4). In UV absorption spectra, it was observed that the size of the alkyl group has only small effects on the shift of the spectrum, though with increasing size, the spectra tend to shift towards longer wavelengths [28]. However, in the obtained REMPI spectra, this shift is not clearly detected for isopropyl compared to methyl groups.

#### 3.2.2 Alkylated PAHs

**FIGURE 5:**
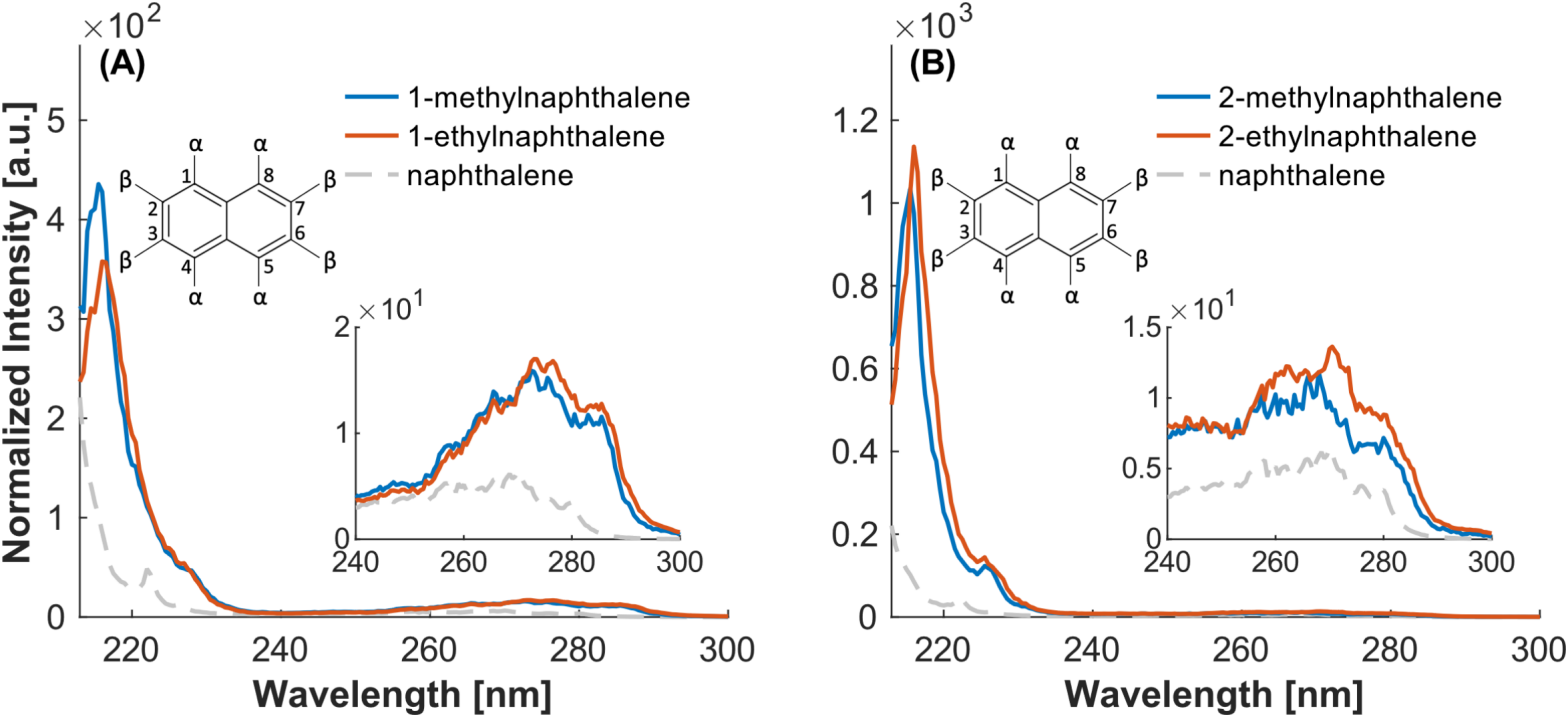
Investigation of the influence of number and position of alkyl groups. (A) Naphthalene substituted on the α‐position. (B) Naphthalene substituted on the β‐position. Intensities normalized to the maximum of toluene.

As described for the alkylated benzenes, the alkylation of naphthalene in general also leads to a bathochromic shift compared to the parent PAH (see Figure 5). It should be noted that in naphthalene there are typically two positions that can be distinguished. The positions closest to the bond between the benzene rings are α‐positions (corresponding to 1, 4, 5, and 8) and the positions farthest away from the bond are β‐positions (corresponding to 2, 3, 6, and 7) [29].

As can be seen in Figure 5, the difference between methyl and ethyl substitution is quite small with respect to the overall shape of the spectrum, as already described in [28]. However, the ethylnaphthalene (EN) is bathochromically shifted by about 0.5 nm with respect to the methylnaphthalene (MN). This is due to the inductive effect being stronger for an ethyl compared to a methyl group [30]. The maximum values for the REMPI spectra are documented in Table 2.

Moreover, a slight bathochromic shift of the 1‐MN/1‐EN spectrum in comparison to the 2‐MN/EN can be observed. This shift has been previously reported by several authors [29, 31, 32] and is due to the higher reactivity of the α‐position, which is a consequence of a higher electron density. The elevated intensity of the β‐substituted naphthalenes can be attributed to the higher lifetime of the excited state [33, 34]. For instance, the S_1_ state of 2‐MN and 2‐EN has a lifetime of 324 ns and 360 ns, respectively, in comparison to 305 ns for 1‐MN [34].

**FIGURE 6:**
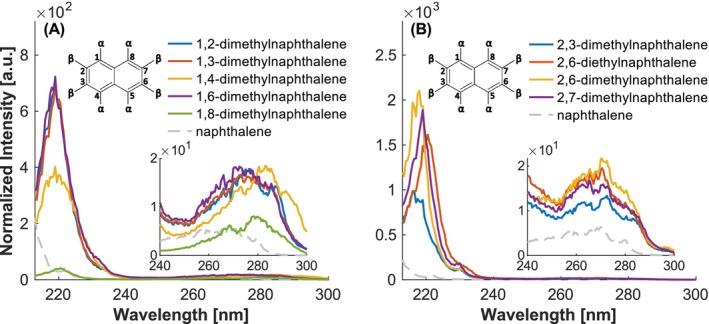
Investigation of the influence of number and position of alkyl groups. (A) DMNs with at least one methyl group at an α‐position. (B) DMNs with methyl groups only on β‐positions. Intensities normalized to the maximum of toluene.

The introduction of a second alkyl group results in an increase of the bathochromic shift. Furthermore, an enhanced bathochromic shift induced by the ethyl group is observed for the 2,6‐diethylnaphthalene (2,6‐DEN). The overall shape of the spectra of the alkylated naphthalenes is quite similar. In comparison to 1,2‐, 1,3‐, and 1,6‐dimethylnaphthalene (DMN), 1,4‐ and 1,8‐DMN exhibit both a hypochromic and bathochromic shift. This can be attributed to the fact that both methyl groups are on α‐positions. The even more pronounced decrease in intensity of 1,8‐dimethylnaphthalene may be attributed to an additional effect of steric hindrance. Conversely, if both methyl groups are placed on β‐positions, the intensity increases accompanied by a hypsochromic shift (see Figure 6).

**FIGURE 7:**
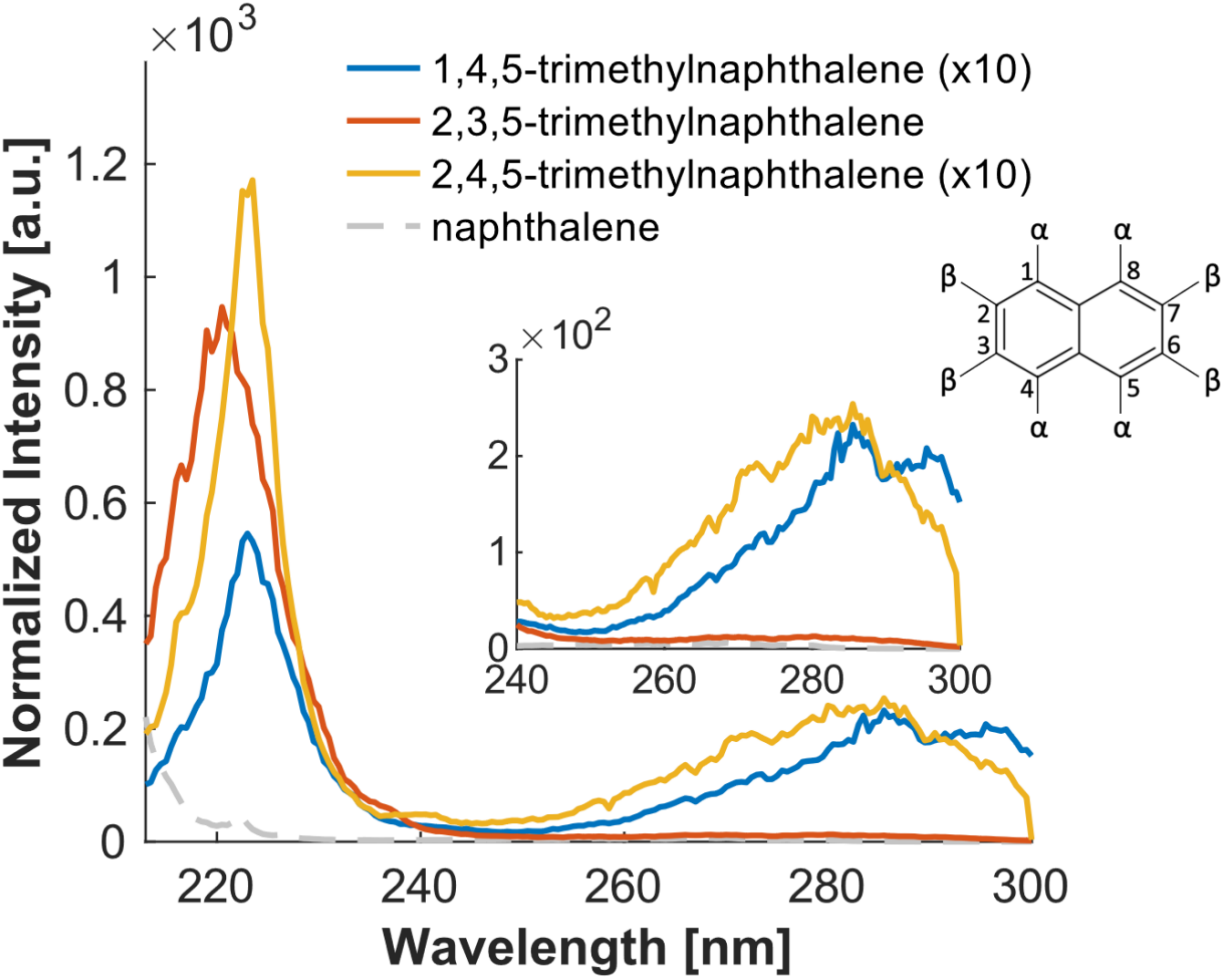
Investigation of the influence of number and position of alkyl groups. TMNs with methyl groups on different positions (α,α,α for 1,4,5‐TMN; β,β,α for 2,3,5 TMN; β,α,α for 2,4,5‐TMN). Intensities normalized to the maximum of toluene.

The same observations can be made for the trimethylnaphthalenes (TMN). The 1,4,5‐TMN substituted only at the α‐position shows the largest bathochromic shift and the lowest intensity. In comparison, 2,3,5‐TMN is shifted towards blue and shows an increased intensity (Figure 7). The effects on the intensity, however, appear to have a considerably greater impact on the S_3_ band around 220 nm than on the S_2_ band observed around 280 nm.

**FIGURE 8:**
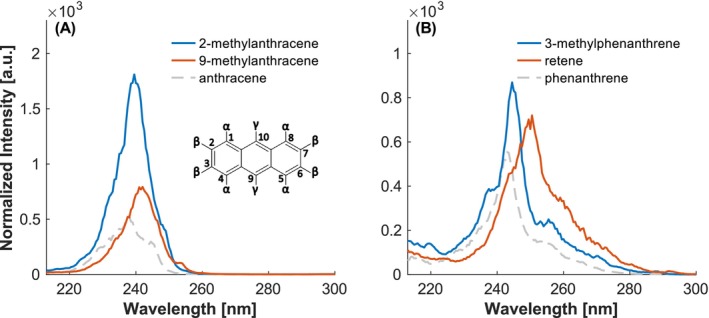
Investigation of the influence of number and position of alkyl groups. (A) Anthracene with a methyl group on a β‐position (2‐methylanthracene) and on a γ‐position (9‐methylanthracene). (B) Phenanthrene with different alkyl groups. Intensities normalized to the maximum of toluene.

The alkylated anthracenes and phenanthrenes exhibit a bathochromic and hyperchromic shift, respectively (see Figure 8). While 2‐methylanthracene is substituted on a β‐position, 9‐methylanthracene represents a γ‐substituted isomer. It is observed that the intensity of the β‐substitution is markedly greater. This phenomenon may be attributed to the enhanced reactivity of the γ‐position [35], as previously documented in naphthalene for the α‐position. The addition of a methyl group alongside an isopropyl group, as in retene, induces a similar effect to that previously observed for cymene. This results in a redshift of the spectrum compared to phenanthrene, with an even more pronounced shift compared to substitution with a single methyl group. However, despite the presence of an additional alkyl group, no hyperchromic effect is observed for retene, which may be attributable to the position of the substituents.

#### 3.2.3 Substituted AHs and PAHs: ‐Cl, ‐SH, and ‐OH

**FIGURE 9:**
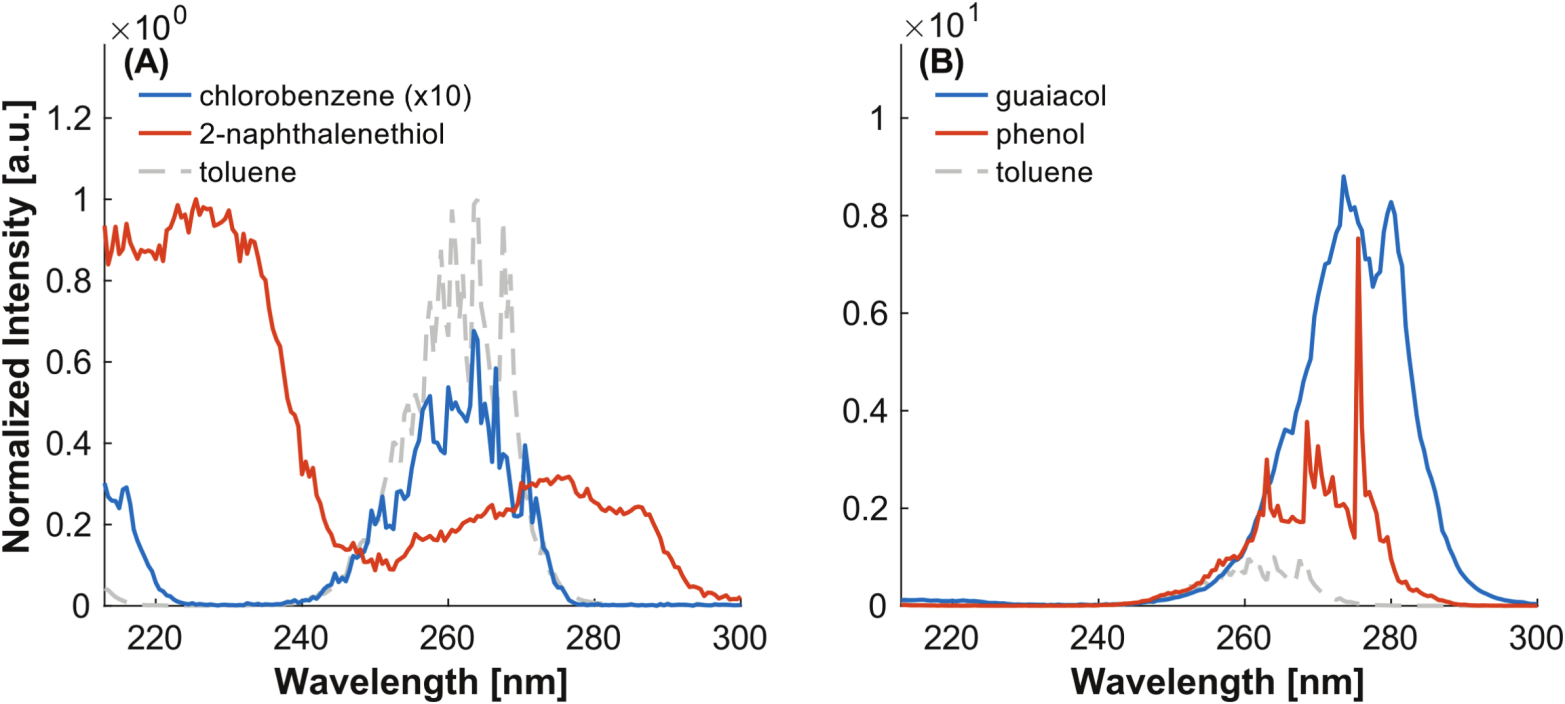
Investigation of the influence of different substituents. (A) Shifts caused by ‐Cl and ‐SH. (B) Shifts caused by CH_3_O and ‐OH. Intensities normalized to the maximum of toluene.

In general, the bathochromic shift caused by the investigated substances is expected to increase in the order of Cl < OH < SH, due to stronger conjugation [36]. A similar behavior can be observed for the REMPI spectra, where phenol is further red‐shifted than chlorobenzene compared to toluene, and the largest shift is observed for the thiol substitution (see Figure 9). However, the latter may be influenced by the number of benzene rings and the position of the substituent. In comparison to the previously examined shifts caused by a methyl group, the shifts caused by SH and OH are significantly higher. Moreover, the thiol‐ and chloride‐substituted compounds show a strong decrease in intensity compared to methyl substitution, which may be attributed to the shorter excited‐state lifetimes of these derivatives. For chlorobenzene and 2‐naphthalenethiol, the S_1_ state lifetimes have been reported to lie in the high picosecond range and low nanosecond range, respectively [37, 38]. Despite the fact that these values were obtained in solution, they provide a reasonable estimation of the order of magnitude, especially when compared to the previously mentioned lifetimes of methylnaphthalenes or the S_1_ lifetime of toluene, which is 86 ns [39]. In contrast, the substitution with ‐OH results in a hyperchromic effect, as observed for phenol and guaiacol. The further increase in redshift for guaiacol can be attributed to the presence of an additional methoxy group.

### 3.3 Heterocyclic Compounds

**FIGURE 10:**
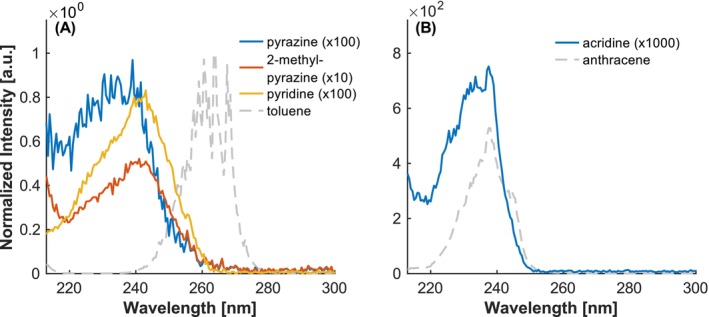
Investigation of the influence of different heteroatoms in six‐membered rings. (A) Shifts caused by nitrogen in monocyclic compounds. (B) Shifts caused by nitrogen in polycyclic compounds. Intensities normalized to the maximum of toluene.

The influence of heteroatom incorporation appears to depend on the size of the ring in which the heteroatom is inserted. For example, replacing a carbon atom with nitrogen in a six‐membered ring—e.g., pyridine, etc.—induces a hypsochromic shift along with a pronounced decrease in absorption intensity (see Figure 10). Both effects become more significant with an increasing number of nitrogen atoms in the ring. The reduced intensity can be ascribed to the shorter lifetimes of the excited states, which have been reported to lie between 0.02 and 0.03 ns for pyridine and pyrazine [40, 41]. For this reason, to obtain reasonable REMPI spectra of those substances, it was necessary to enhance the photon density by utilizing a lens (f = 200 mm) within the laser beam path. The lens was positioned in a manner that the ionization occurred off‐focus, as an excessively high photon density would have resulted in fragmentation or saturation. As a result, the diameter of the laser beam was reduced to approximately 1 mm. Consequently, a comparison to the other spectra is only possible to a limited degree and should be restricted to the overall shape of the spectra.

**FIGURE 11:**
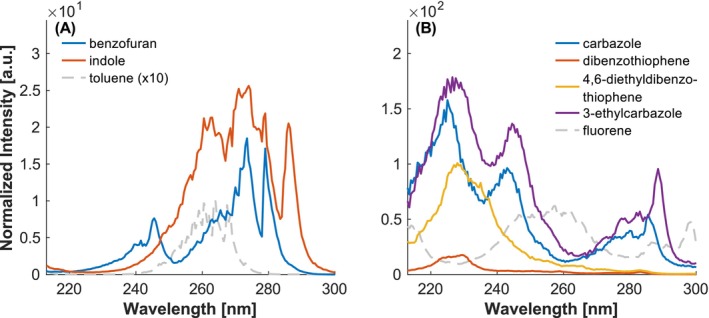
Investigation of the influence of different heteroatoms in five‐membered rings. (A) Shifts caused by oxygen (benzofuran) and nitrogen (indole) in five‐membered rings of bicyclic compounds. (B) Shifts caused by nitrogen (carbazole) and sulfur (dibenzothiophene) in five‐membered rings in tricyclic compounds. Intensities normalized to the maximum of toluene.

With respect to the spectra depicted in Figure 11A, it is important to note that, while the overall spectra are comparable to those of toluene, the addition of a five‐membered ring induces a significant bathochromic shift, as previously discussed in Section 3.1. This shift is attributed to the presence of an additional conjugated double bond, as reported in [36]. In general, for all of the investigated heteroatoms (O, N, S), a blueshift compared to the respective parent PAHs is expected, with the shift for nitrogen being larger than for oxygen [36, 42]. Consequently, the bands of indole are positioned at longer wavelengths than those of benzofuran, which can be attributed to the stronger delocalization of the electrons [43]. In agreement with the observations in literature [44], carbazole and dibenzothiophene also show a blue shift relative to fluorene, see Figure 11B.

In contrast to the nitrogen in the six‐membered rings, an increase in intensity is recorded for PAHs with five‐membered rings containing nitrogen, such as indole and carbazole. This behavior is also observed for oxygen. Conversely, for sulfur‐containing systems, such as dibenzothiophene, intensity decreases similarly to the previously discussed substitution in naphthalenethiol, though not to the same extent as observed in six‐membered rings. However, regardless of the size of the ring, the same effects as previously reported, such as a redshift due to alkylation (e.g., 2‐methylpyrazine, 4,6‐dibenzothiophene, 3‐ethylcarbazole), can be observed for substituents.

In general, it can be seen that the overall spectral shape differs significantly between each aromatic core structure, with linear annellation leading to large bathochromic shifts. This allows the assignment of structural and chemical information to masses in a nominal mass spectrum by differentiating between isobaric hetero and non‐hetero PAHs (i.e., dibenzothiophene and diethylnaphthalene) and even isomeric core structures (i.e., anthracene and phenanthrene) by switching through suitable wavelengths. Different substituents and substitution patterns also lead to shifts in the spectra, such as the bathochromic shift through alkylation, although to a lesser extent than differences in the core structure, with the first substitution having a stronger effect than subsequent ones. As the overall spectral shape with its distinctive features of the underlying core structure is usually preserved, an attribution to the respective structure is still possible and, in some cases, even a distinction between some substitution isomers is achievable. The investigations have also shown the dependency of the intensity on the lifetimes of the excited states. It has been demonstrated that molecules for which the excited‐state lifetimes are significantly shorter than the pulse width of the laser (i.e., pyridine) exhibit substantially lower intensities. Consequently, for a practical application, it may be advantageous to employ a laser with a shorter pulse width (i.e., picosecond lasers) for those molecules to enhance the sensitivity [45].

## 4 Cross Sections

For a quantification of the measured spectra, the calculation of relative photoionization cross sections (relPICS) has been proven to be a promising approach [46]. For this purpose, the response factor of the analyte (*r*_*A*_) is set in relation to the response factor of a reference substance (*r*_*ref*_). The response factor is defined as the ratio between the integrated signal intensity and the concentration of the analyte. These response factors can then be used to calculate relPICS (*σ*_*rel,ref*_):

(1) $$\sigma_{\mathrm{rel}}=\frac{r_{A}}{r_{\mathrm{ref}}}*I^{\left(n_{\mathrm{ref}}-n_{A}\right)}$$

where *n* refers to exponents of analyte (*A*)/reference (*ref*) and *I* refers to laser intensity.

A more detailed description of the calculation of relPICS can be found in Gehm et al. [46]. As can be concluded from Equation (1), the relPICS are influenced by the exponents n_A_ and n_ref_, which represent the exponents obtained by plotting the signal intensity against the laser intensity (see Supporting Information).

REMPI is a process that basically requires two steps, of which (a) the absorption of a photon and excitation of the molecule is followed by (b) the absorption of a second photon and ionization. The probability of both steps occurring is expressed by the cross section *σ*_1_ for (a) and *σ*_2_ for (b). For an unfocused laser beam and if no saturation effects occur, a quadratic dependence of the signal intensity on the applied laser energy can be observed (for *σ*_1_ ≈ *σ*_2_). For higher laser energies, saturation causes the intensity to transition from a quadratic to a linear dependence [47]. If geometrical effects of the laser beam and saturation effects are taken into account, a behavior of *S* ~ *I* ^2−*x*^ (*S*, signal intensity; *I*, laser intensity; *x*, correction parameter) is generally expected [6].

In order to determine the exponents *n*_*A*_ and *n*_*ref*_, laser energies in the range of 20–60 μJ were applied to several substances with different numbers of rings and alkyl groups. The measurements were carried out for two commonly applied wavelengths of non‐tunable lasers, 248 and 266 nm, and *λ*_*max*_, which was selected because saturation effects are most likely to occur [47].

This allows the exponents and potential deviations from quadratic dependence to be evaluated. In order to perform more accurate calculations of relPICS in the future, the measurements must be carried out for each substance at all wavelengths of interest. As can be observed in Table 1, most of the determined exponents suggest a quadratic behavior and hence no saturation effects for the applied energy range. This outcome validates the selected energy range for the investigation of relPICS and should also ensure that the spectral shape is independent from the laser energy in this range, making it reasonable to calculate relPICS not only for other compounds but also for other wavelengths (e.g., 213 nm). In consideration of the obtained data and potential measurement inaccuracies, it was reasonable to assume that *n*_*A*_ ≈ *n*_*ref*_.

**TABLE 1:** Exponents gained from plotting laser energy against intensity (see Supporting Information).

	*n* (248 nm)	*n* (266 nm)	*n* (λ_max_)
Toluene	1.9	1.9	2.0
o‐Xylene	2.0	2.0	2.0
p‐Cymene	2.0	1.8	1.9
Naphthalene	2.0	2.0	2.1
1‐MN	2.0	1.8	2.0
2,7‐DMN	1.8	2.0	2.0
Anthracene	1.9	—	1.9
Phenanthrene	2.0	2.0	2.0
Fluorene	2.0	2.0	2.0
Pyrene	2.0	2.0	2.0

Hence, Equation (1) can be written as

(2) $$\sigma_{\mathrm{rel}}\approx \frac{r_{A}}{r_{\mathrm{ref}}}$$

With that, *σ*_*rel,tol*_ was calculated for all PAHs at 213 nm (fifth harmonic of a Nd:YAG laser), 248 nm (output wavelength of a KrF excimer laser), and 266 nm (fourth harmonic of a Nd:YAG laser) as well as *λ*_*max*_ (see Table 2). The given *λ*_*max*_ represents the absolute maxima of the spectra. It should be noted that the peaks of the (P)AHs are typically extended over a range of several nanometers. The obtained data for *σ*_*rel,tol*_ (248 nm) and *σ*_*rel,tol*_ (266 nm) were compared to the data given in [46] and showed good correlations for the substances investigated in the previous study. It is evident from Table 2 that most of the analyzed aromatics have a higher response factor than toluene at the wavelengths investigated. Especially the PAHs, whose response factor is comparatively high even for wavelengths at which their spectra show only weak bands. At their specific *λ*_*max*_, the response factors can exceed that of toluene by up to six orders of magnitude. The single ring benzene derivatives usually exhibit a lower relPICS at 248 nm than at 266 nm, often below a value of one, due to their redshift compared to the toluene band. Of the chosen commonly applied laser wavelengths, 213 nm appears to be particularly beneficial for most of the substances, especially for the naphthalenes, which proves the importance of the measurements down to such low wavelengths.

**TABLE 2:** Calculated relPICS for the investigated compounds with toluene as reference.

Substances	*λ*_*max*_ [nm]	*σ*_*rel,tol*_ (213 nm)	*σ*_*rel,tol*_ (248 nm)	*σ*_*rel,tol*_ (266 nm)	*σ*_*rel,tol*_ (*λ*_*max*_)
1‐EN	216	5.32E+03	33.1	24.3	5.72E+04
1,2,3‐TMB	272.5	2.7	0.2	1.6	10.7
1,2,4‐TMB	275.5	4.8	0.8	2.9	55.5
1,2‐DMN	219	7.89E+03	41.2	36.0	1.48E+06
1,3‐DMN	219	7.94E+03	42.2	32.1	1.42E+06
1,4,5‐TMN	223	249	11.6	18.2	1.70E+05
1,4‐DMN	219	5.60E+03	26.3	27.3	8.21E+05
1,6‐DMN	219	8.88E+03	45.6	45.6	1.46E+06
1,8‐DMN	220.5	383	9.3	25.3	4.73E+04
1‐MN	215.5	7.04E+03	38.3	34.3	7.40E+04
2,3,5‐TMN	220.5	8.22E+03	67.7	27.6	1.55E+06
2,3‐DMN	216.5	3.96E+04	90.4	34.2	2.01E+05
2,4,5‐TMN	223.5	469	23.3	25.5	4.02E+05
2,6‐DEN	220.5	1.56E+04	89.5	26.9	5.93E+06
2,6‐DMN	218	3.32E+04	79.1	51.0	1.33E+06
2,7‐DMN	219	2.31E+04	67.0	33.8	3.84E+06
2‐EN	216	1.15E+04	56.3	29.1	1.82E+05
2‐Methylanthracene	239.5	1.11E+03	2.76E+03	11.1	2.56E+05
2‐MN	215.5	1.61E+04	53.7	29.8	1.38E+05
3‐Methylphenanthrene	244.5	6.68E+03	3.43E+03	268	2.22E+04
9‐Methylanthracene	242	512	2.03E+03	10.0	3.52E+04
Acenaphthylene	219.5	11.1	0.01	0.08	427
Acridine	237.5	17.3	0.3	—	171
Anthracene	238	651	863	4.8	1.92E+05
Benzofuran	273.5	5.9	19.9	11.9	139
Carbazole	225	2.85E+03	485	45.2	2.12E+05
Dibenzothiophene	229.5	164	28.1	4.7	9.13E+04
4,6‐Diethyldibenzo‐thiophene	228	1.43E+03	152	16.6	6.31E+04
3‐Ethylcarbazole	226.5	1.93E+03	694	51.7	5.74E+05
Fluorene	257.5	1.28E+03	328	106	95
Indole	274	16.4	28.5	27.2	268
m‐Xylene	271.5	1.9	0.4	1.7	7.5
Naphthalene	213	8.23E+03	40.0	22.2	8.23E+03
2‐Naphthalenethiol	225.5	272.7	8.5	2.9	2.58E+04
o‐Xylene	270	1.8	0.7	1.7	3.6
Phenanthrene	243	2.54E+03	2.31E+03	188	3.20E+04
Phenol	275.5	0.2	0.8	2.5	204
p‐Xylene	267	5.2	1.7	2.3	2.3
Pyrene	232.5	1.55E+03	1.06E+03	451	1.83E+06
Retene	250.5	3.09E+03	4.04E+03	677	3.62E+03
Chlorobenzene	263.5	0.6	0.07	0.05	0.07
o‐Cymene	270.5	0.4	0.3	0.8	1.7
p‐Cymene	272	3.1	1.6	2.5	12
Guaiacol	273.5	1.0	0.4	3.5	28.8

*Note:* The relPICS for pyridine, pyrazine, 2‐methylpyrazine, and acridine were not calculated due to the deviating optical setup.

In the context of the present work, the demonstrated REMPI spectra have been mostly evaluated with regard to analogous structures. Hence, the calculation of relPICS is a useful tool for comparison among all of the measured data. While cross sections of *λ*_*max*_ primarily function as a general indicator of the ionization efficiency, the calculation of *σ*_*rel,tol*_ (213 nm), *σ*_*rel,tol*_ (248 nm), and *σ*_*rel,tol*_ (266 nm) is particularly advantageous for the selection of a suitable non‐tunable laser. Together with the information obtained by REMPI spectroscopy, these findings can not only help to identify potential health‐relevant PAHs in complex matrices but also quantify them against readily and cheaply available standards.

## 5 Conclusion

In the presented study, we have recorded (1 + 1)‐REMPI spectra of pure AHs and PAHs via REMPI‐TOFMS in a wavelength range from 213 to 300 nm. The obtained spectra of several substances were compared to data given in the literature and verified the applicability of this technique. Further comparison had been made with absorption spectra and showed a good agreement with reported absorption bands. Spectral shifts were observed to occur in dependency on ring number, annellation, and substituents.

Based on the spectral data, the relPICS were calculated with the objective of achieving an instrument‐independent quantification of the compounds. In order to do so, the response factors of the substances were compared to that of toluene, expressing the relPICS as the probability of the ionization process relative to toluene. It was observed that the applied wavelength region was particularly advantageous for the naphthalenes, showing comparably high relPICS at 213 nm and exhibiting maxima typically below 220 nm. Besides the identification of the PAHs through their spectra, the measured relPICS allow for their quantification against an internal standard, which is equally vital to estimate their toxicological potential.

The results demonstrate that differences in size and heteroatom count of the aromatic system result in REMPI spectra sufficiently distinct to facilitate the identification of substances within a complex sample. Spectral shifts recorded for isomers with varying substituent positions are relatively minor. Therefore, differentiation of isomers is possible when electron densities differ between substituent positions. Furthermore, as the overall band structure of the aromatic core is usually preserved, except for the shift in position, structural insight should still be gained even for substitution patterns that have not been recorded previously. In future work, the data obtained will be used to investigate complex samples. This will involve switching between at least two wavelengths to differentiate between isomers.

While the spectra in this study were recorded over a period of approx. 25 min each, with a resolution of 0.5 nm, the broad bands spanning often tens of nanometers allow the distinction of core structures and isomers at much reduced resolutions and thus shorter measurement times. With the use of fast scanning OPOs, a complete spectrum with 5 nm resolution could theoretically be acquired in just over 1 s, although averaging over several scans would likely still be needed for better spectral quality. Nonetheless, this would bring the spectra acquisition time down to a timeframe that is applicable in the on‐line analysis of slowly evolving gas mixtures such as thermogravimetry or the measurement of combustion profiles and would give photoionization mass spectrometry an additional capability to identify health relevant PAHs in mixtures. An integration of such a fast‐switching OPO into a thermogravimetric analyzer or a thermal‐optical carbon analyzer will be explored in future works.

## Author Contributions

**Carolin Schwarz:** writing – original draft, methodology, investigation, visualization. **Fabian Etscheidt:** writing – original draft, software, methodology. **Christian Gehm:** conceptualization, methodology, writing – review and editing. **Johannes Passig:** methodology, writing – review and editing. **Sven Ehlert:** writing – review and editing. **Thorsten Streibel:** supervision, writing – review and editing. **Ralf Zimmermann:** funding acquisition, writing – review and editing.

## Conflicts of Interest

The authors declare no conflicts of interest.

## Peer Review

The peer review history for this article is available at https://www.webofscience.com/api/gateway/wos/peer‐review/10.1002/rcm.10096.

## Supporting information


**Figure S1** Investigation of the influence of the size of alkyl groups. (A) Ortho‐xylene, containing two methyl groups, and ortho‐cymene, containing one methyl and one isopropyl group. (B) Para‐xylene, containing two methyl groups, and para‐cymene, containing one methyl and one isopropyl group.
**Figure S2.** Investigation of the influence of the position of alkyl groups. (A) Methyl‐substituted naphthalene on the α‐position (1‐MN) compared to the β‐position (2‐MN). (B) Ethyl‐substituted naphthalene on the α‐position (1‐EN) compared to the β‐position (2‐EN).
**Figure S3.** The dependence of the intensity on the laser energy for several compounds in the wavelength range from 20 to 60 μJ at a wavelength of 248 nm for the determination of the exponents for the calculation of the relPICS.
**Figure S4.** The dependence of the intensity on the laser energy for several compounds in the wavelength range from 20 to 60 μJ at a wavelength of 266 nm for the determination of the exponents for the calculation of the relPICS.
**Figure S5.** The dependence of the intensity on the laser energy for several compounds in the wavelength range from 20 to 60 μJ at λ_max_ for the determination of the exponents for the calculation of the relPICS.

## Data Availability

Data will be made available on request.
