# Supplementary material for: Resonance‐Enhanced Multiphoton Ionization Spectroscopy of Monocyclic and Polycyclic Aromatic Hydrocarbons in the Gas Phase
Source: Rapid Commun Mass Spectrom. 2025 Jun 21;39(19):e10096. doi: 10.1002/rcm.10096 (PMC12181801; doi:10.1002/rcm.10096)
Supplement: Supplementary file 1 — Figure S1 Investigation of the influence of the size of alkyl groups. (A) Ortho‐xylene, containing two methyl groups, and ortho‐cymene, containing one methyl and one isopropyl group. (B) Para‐xylene, containing two methyl groups, and para‐cymene, containing one methyl and one isopropyl group. Figure S2. Investigation of the influence of the position of alkyl groups. (A) Methyl‐substituted naphthalene on the α‐position (1‐MN) compared to the β‐position (2‐MN). (B) Ethyl‐substituted naphthalene on the α‐position (1‐EN) compared to the β‐position (2‐EN). Figure S3. The dependence of the intensity on the laser energy for several compounds in the wavelength range from 20 to 60 μJ at a wavelength of 248 nm for the determination of the exponents for the calculation of the relPICS. Figure S4. The dependence of the intensity on the laser energy for several compounds in the wavelength range from 20 to 60 μJ at a wavelength of 266 nm for the determination of the exponents for the calculation of the relPICS. Figure S5. The dependence of the intensity on the laser energy for several compounds in the wavelength range from 20 to 60 μJ at λmax for the determination of the exponents for the calculation of the relPICS. [file RCM-39-e10096-s001.docx]

**Supporting** **Information** **For:**

**Resonance-Enhanced Multiphoton Ionization Spectroscopy of Mono- and Polycyclic Aromatic Hydrocarbons in the Gas Phase**

Carolin Schwarz^1,2^, Fabian Etscheidt^1,3^, Christian Gehm^1,2^, Johannes Passig^1,4,5^, Sven Ehlert^3^, Thorsten Streibel^1,5*^, Ralf Zimmermann^1,4,5^

1 – Joint Mass Spectrometry Centre / Chair of Analytical Chemistry, University of Rostock, 18059 Rostock, Germany

2 – Leibniz Institute for Baltic Sea Research Warnemünde, 18119 Rostock, Germany

3 – Photonion GmbH, 19061 Schwerin, Germany

4 – Department Life, Light & Matter (LLM), University of Rostock, 18059 Rostock, Germany

5 – Joint Mass Spectrometry Centre, Cooperation Group “Comprehensive Molecular Analytics” (CMA), Helmholtz Zentrum München, Neuherberg, 85764, Germany

* – corresponding author: thorsten.streibel@uni-rostock.de

**Keywords:** resonance-enhanced multiphoton ionization (REMPI), time-of-flight mass spectrometry (TOFMS), spectroscopy, (polycyclic) aromatic hydrocarbons ((P)AH), optical parametric oscillator (OPO)

Table of Contents:

| Figure S1 | Investigation of the influence of the size of alkyl groups. | Page 2 |
| --- | --- | --- |
| Figure S2 | Investigation of the influence of the position of alkyl groups. | Page 2 |
| Figure S3 | The dependence of the intensity on the laser energy at 248 nm. | Page 3 |
| Figure S4 | The dependence of the intensity on the laser energy at 266 nm. | Page 3 |
| Figure S5 | The dependence of the intensity on the laser energy at λ_max_. | Page 4 |


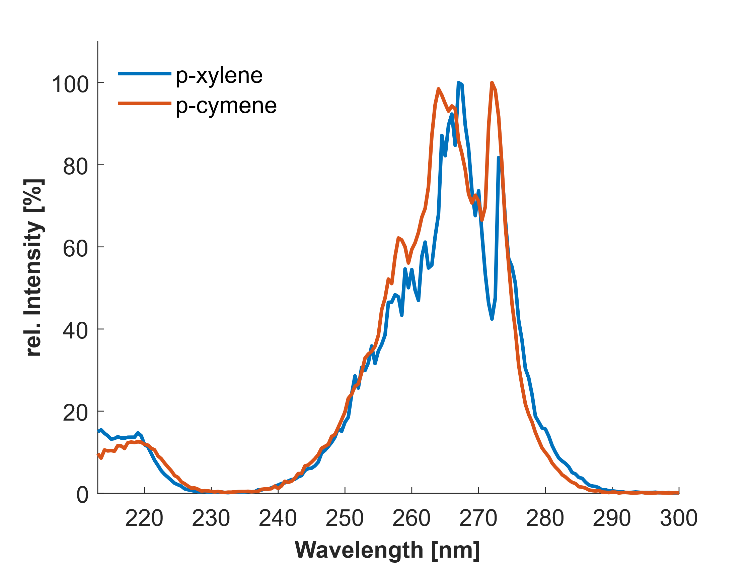

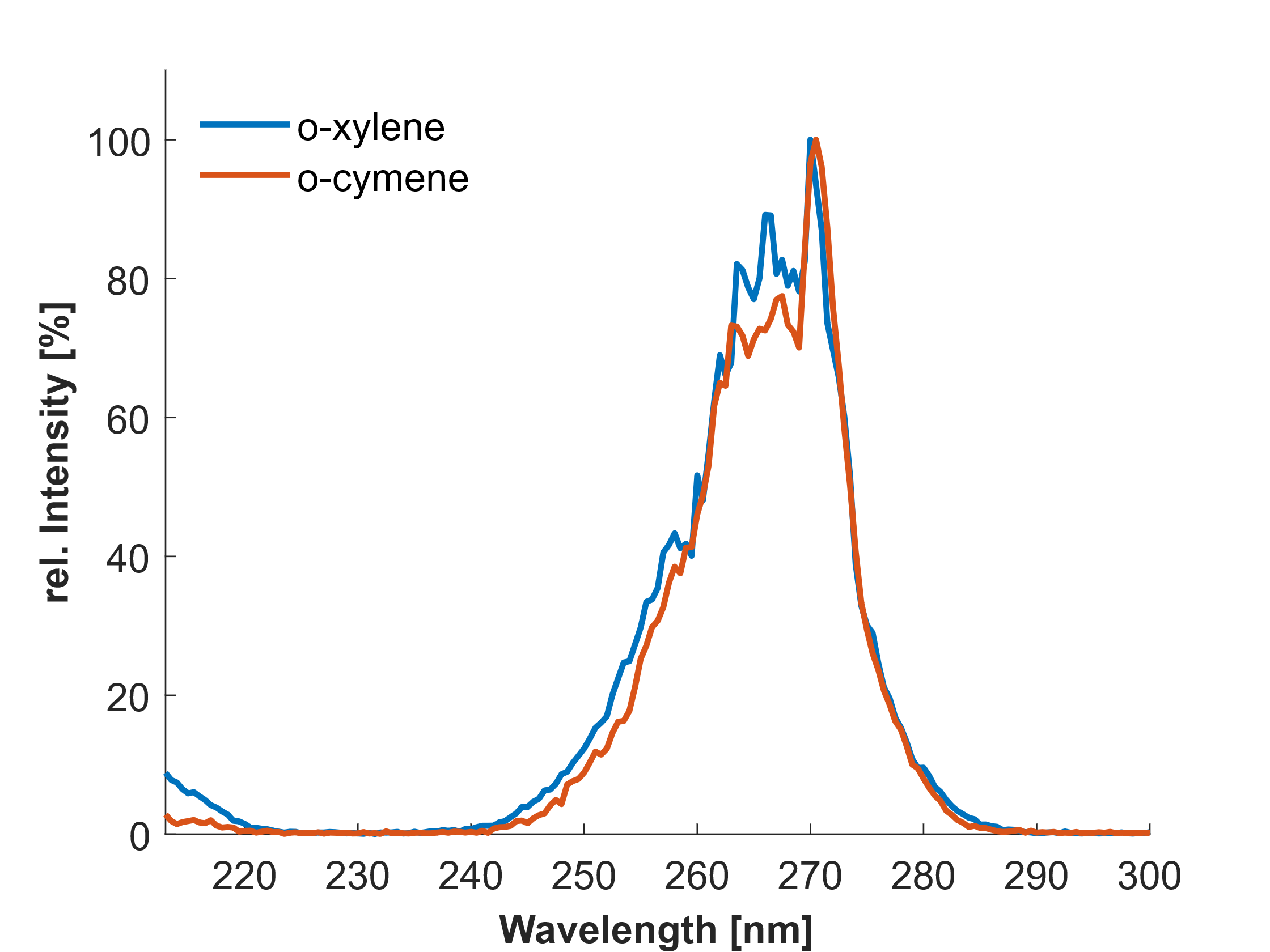


Figure S1 Investigation of the influence of the size of alkyl groups. (A) Ortho-xylene, containing two methyl groups, and ortho-cymene, containing one methyl and one isopropyl group. (B) Para-xylene, containing two methyl groups, and para-cymene, containing one methyl and one isopropyl group.


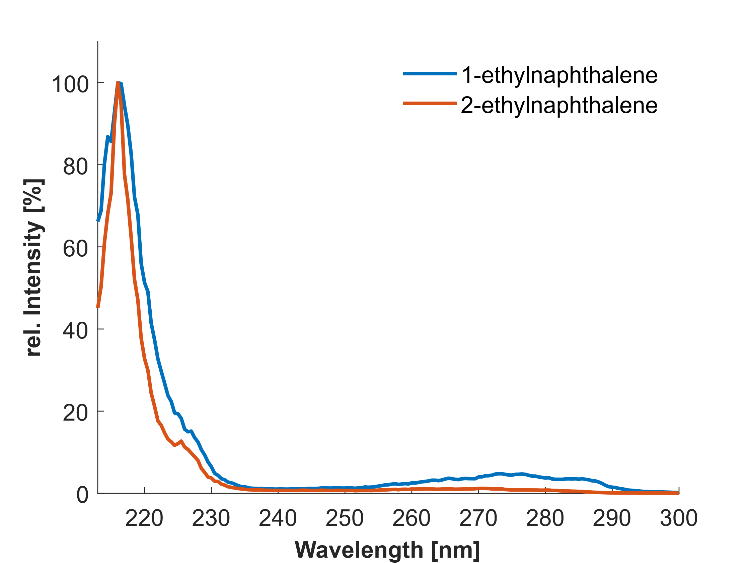

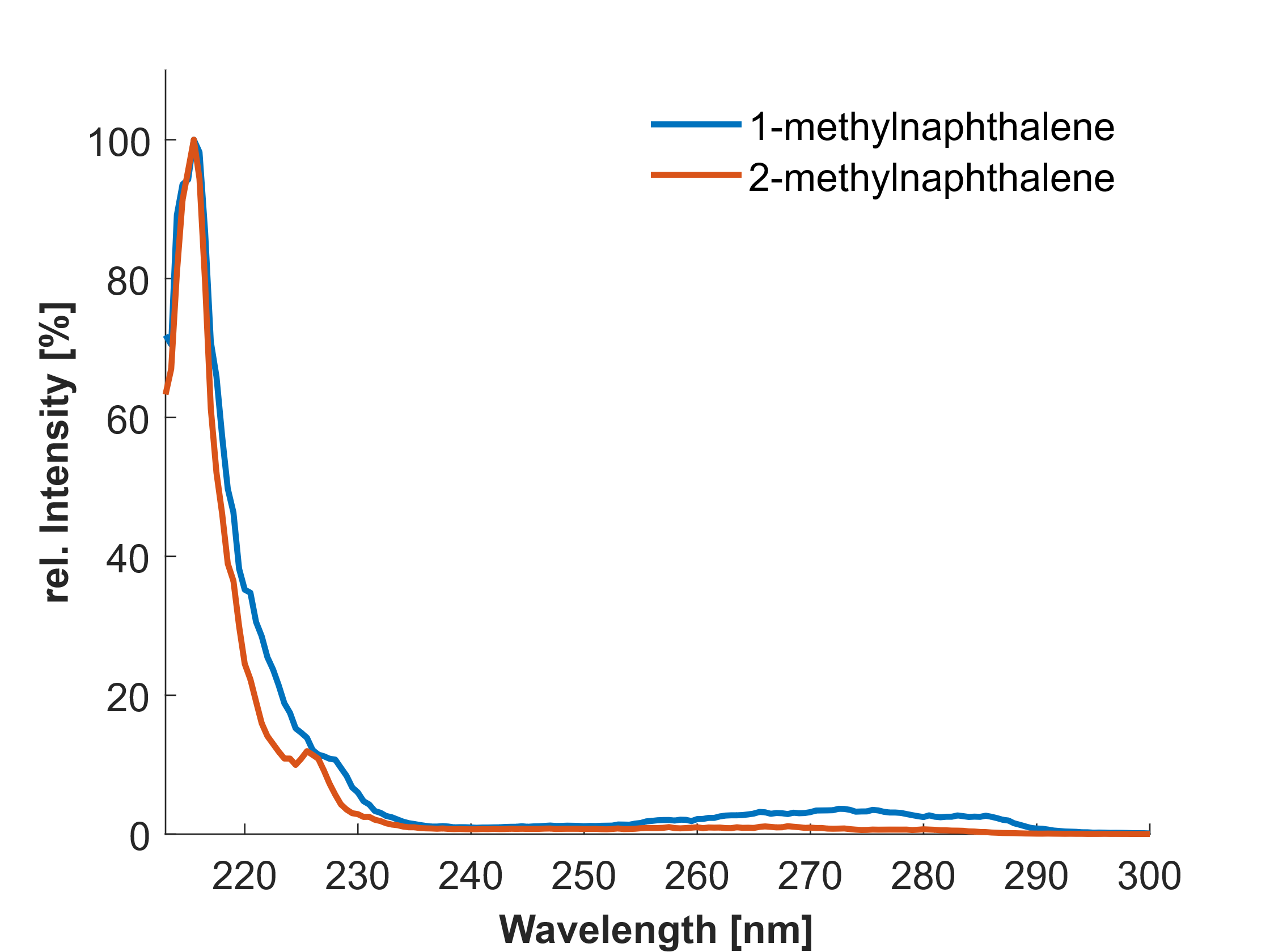


Figure S2 Investigation of the influence of the position of alkyl groups. (A) Methyl-substituted naphthalene on the α-position (1-MN) compared to the β-position (2-MN). (B) Ethyl-substituted naphthalene on the α-position (1-EN) compared to the β-position (2-EN).


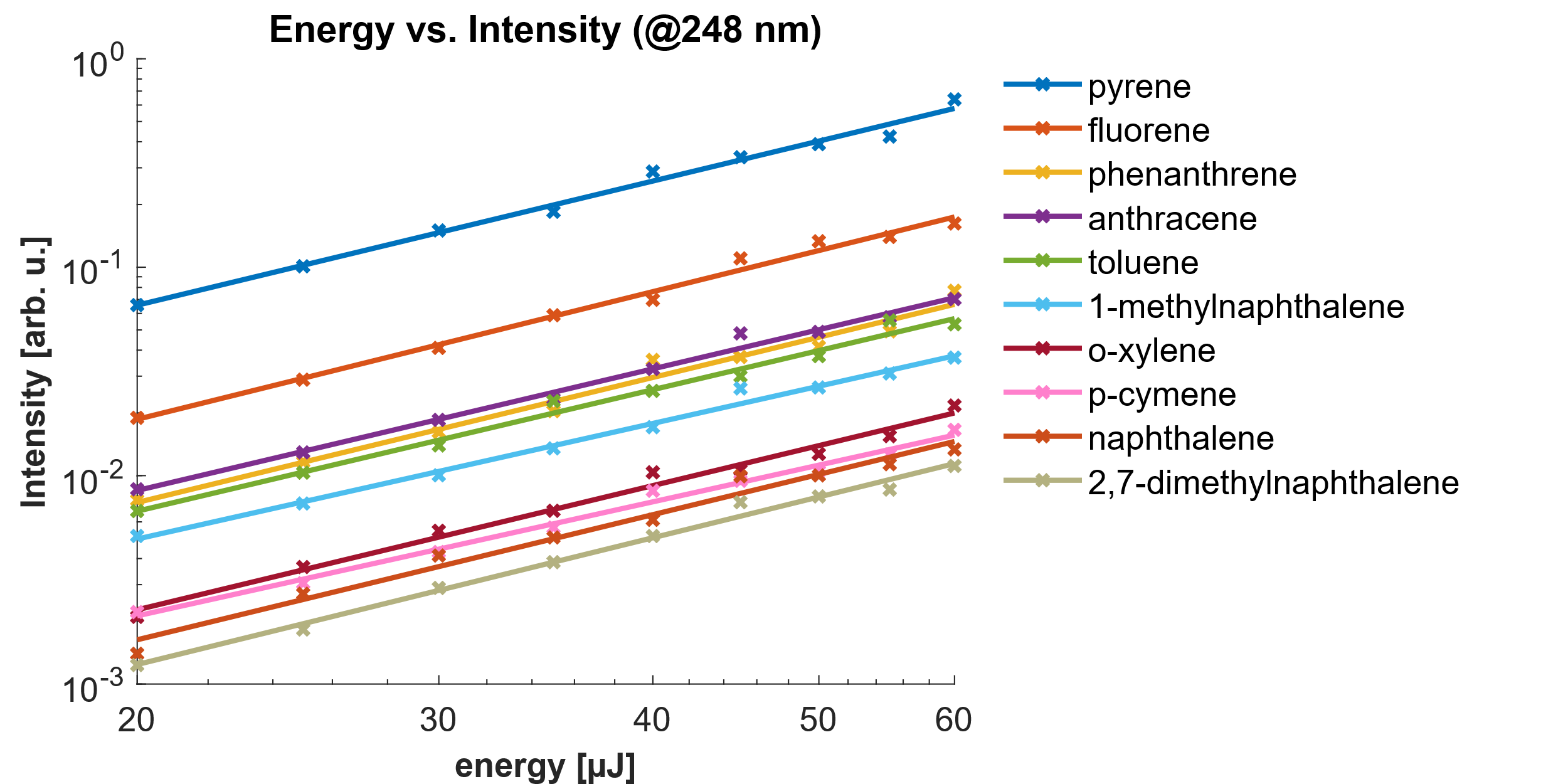


Figure S3 The dependence of the intensity on the laser energy for several compounds in the wavelength range from 20 to 60 µJ at a wavelength of 248 nm for the determination of the exponents for the calculation of the relPICS.


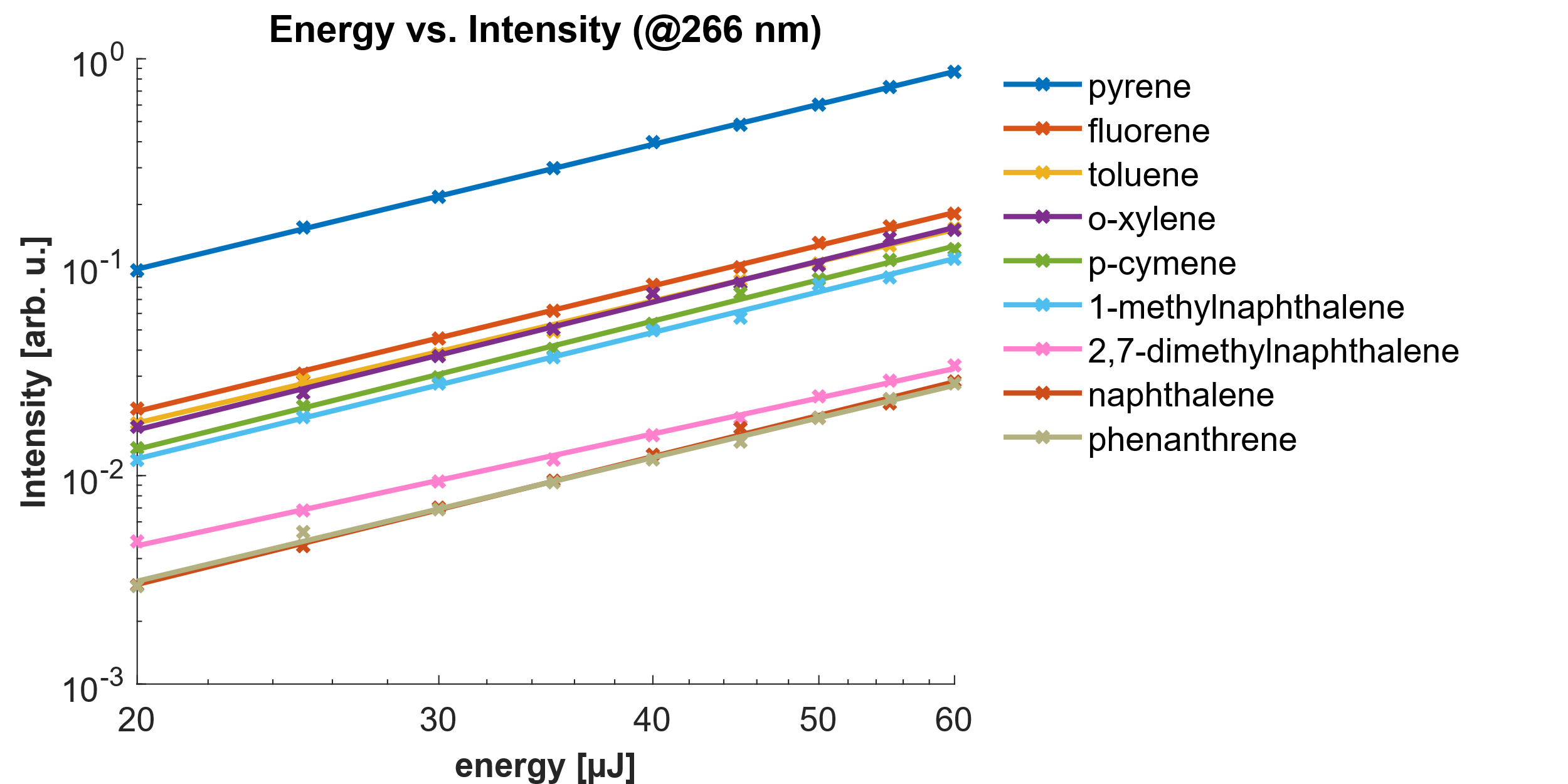


Figure S4 The dependence of the intensity on the laser energy for several compounds in the wavelength range from 20 to 60 µJ at a wavelength of 266 nm for the determination of the exponents for the calculation of the relPICS.


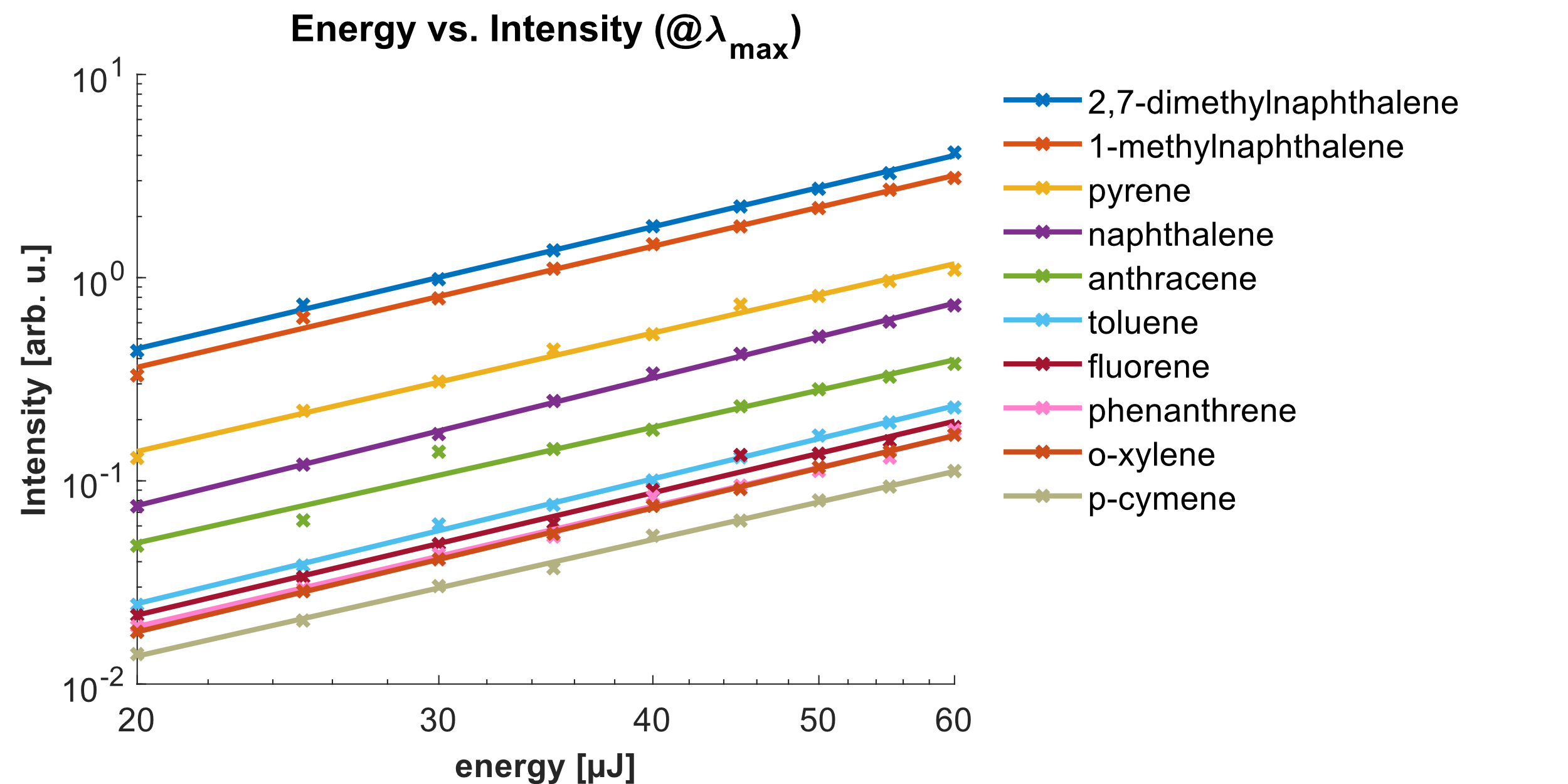


Figure S5 The dependence of the intensity on the laser energy for several compounds in the wavelength range from 20 to 60 µJ at λ_max_ for the determination of the exponents for the calculation of the relPICS.
